# Supplementary material for: Towards colloidal spintronics through Rashba spin-orbit interaction in lead sulphide nanosheets
Source: Nat Commun. 2017 Jun 7;8:15721. doi: 10.1038/ncomms15721 (PMC5467232; doi:10.1038/ncomms15721)
Supplement: Supplementary Information — Supplementary Figures and Supplementary References [file ncomms15721-s1.pdf]

Supplementary Figures

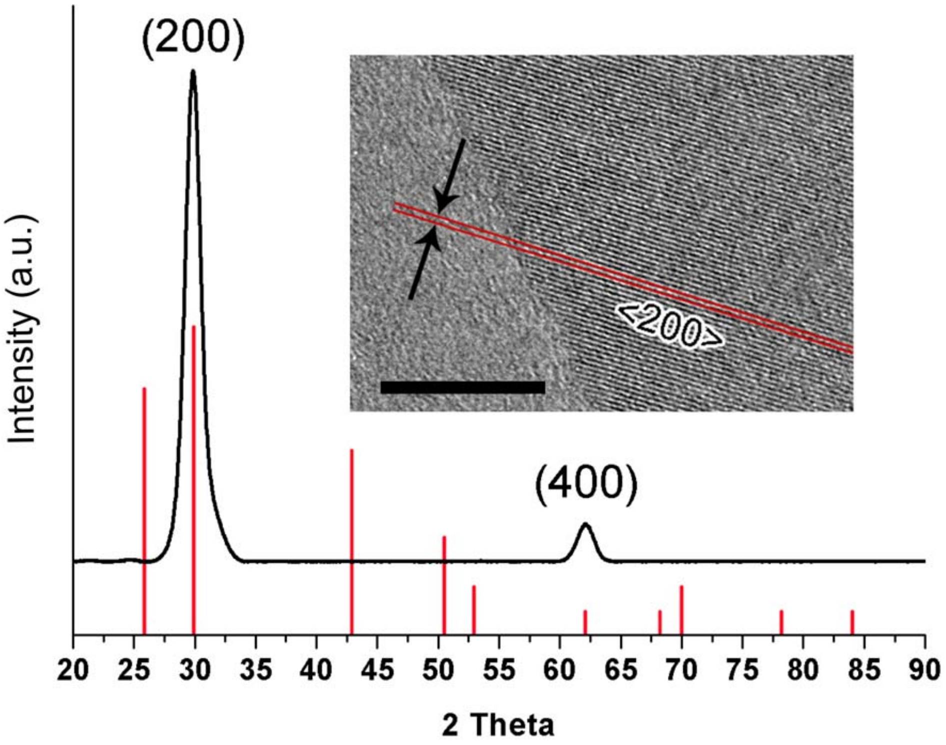

Supplementary Figure 1 | Crystallography of the lead sulfide nanosheets.

X-ray powder diffraction of PbS nanosheets shows only the (200) and (400) planes due to texture effects. The peaks for bulk *galena* are shown in red (JCPDS 5–592: *galena*). The *d*-spacing for the (200) bulk PbS plane is $d = 0.2969$ nm (JCPDS 5–592: *galena*) while the measured *d*-spacing in the high resolution tunnelling-electron microscope (HRTEM) inlet of the (200) nanosheet plane (red lines enclosed by the black arrows) is $d = 0.302 \pm 0.02$. The scale bar is equal to 10 nm.

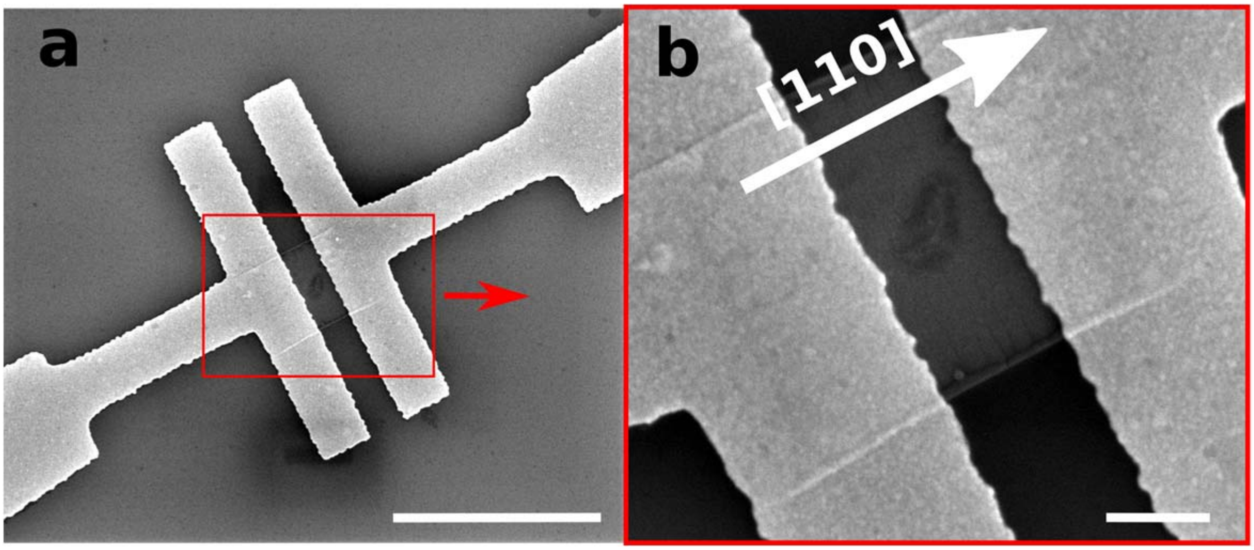

Supplementary Figure 2 | Scanning-electron microscope image of a device based on an individual nanosheet.

a, An SEM image of an individually contacted nanosheet. The scale bar is 4 µm. The nanosheets with thickness of 6, 9 and 18 nm were distributed on the Si/SiO_2 substrate and contacted by Ti/Au by means of electron-beam lithography. **b**, The magnified image of the same device (the scale bar is 500 nm). The current flow for these nanosheets is in [110] direction.

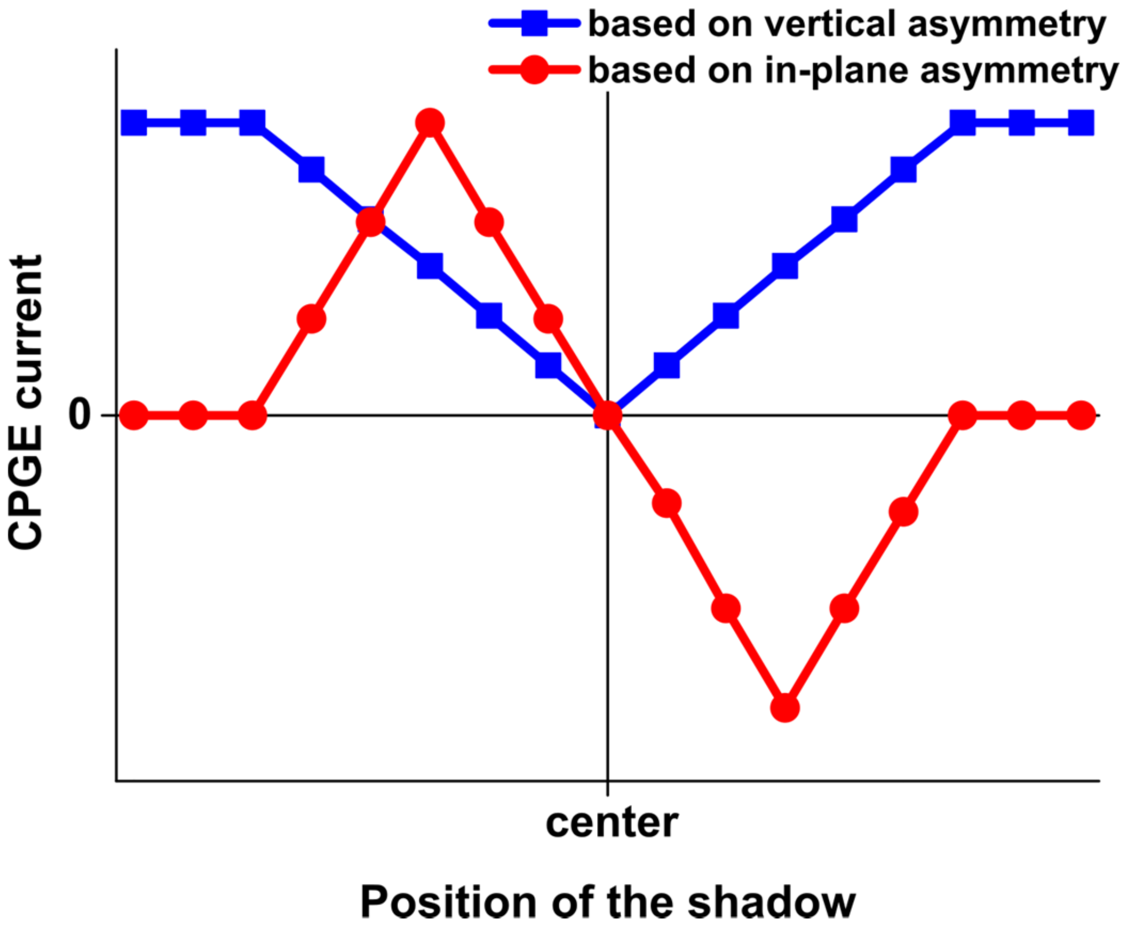

Supplementary Figure 3 | Ideal contribution of vertical and in-plane asymmetry in circular photo-galvanic effect.

If only an in-plane asymmetry is considered, by shadowing the device, the CPGE current experiences a sign change, when the asymmetry is reversed, but the magnitude of the current remains constant. In case of the vertical asymmetry, shadowing leads to a decrease of the CPGE current without changing the sign, since the direction of the asymmetry is constant and the effective part of the crystal for the current generation is reduced. The combination of both effects results in CPGE in PbS nanosheets, while the contribution of each part can be altered by the incidence angle.

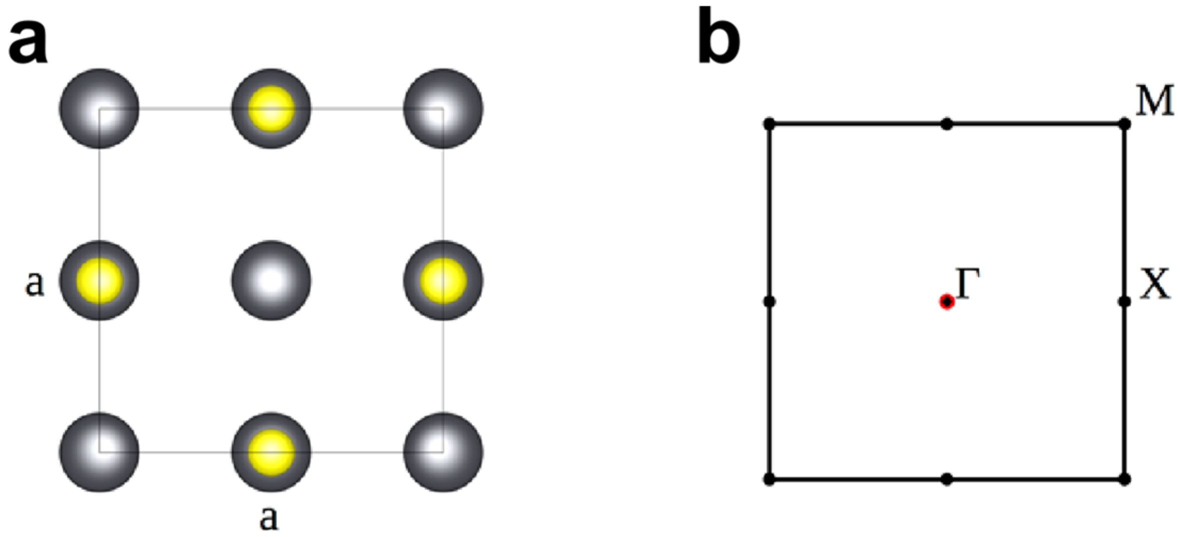

Supplementary Figure 4 | Used unit cell and the corresponding Brillouin zone.

a, The used unit cell during the calculations where *a* denotes the lattice constant and the circles show lead and sulphur atoms. **b**, The corresponding Brillouin zone is shown. The band structure was calculated along the Γ-M-X path and around to the band gap (M Point).

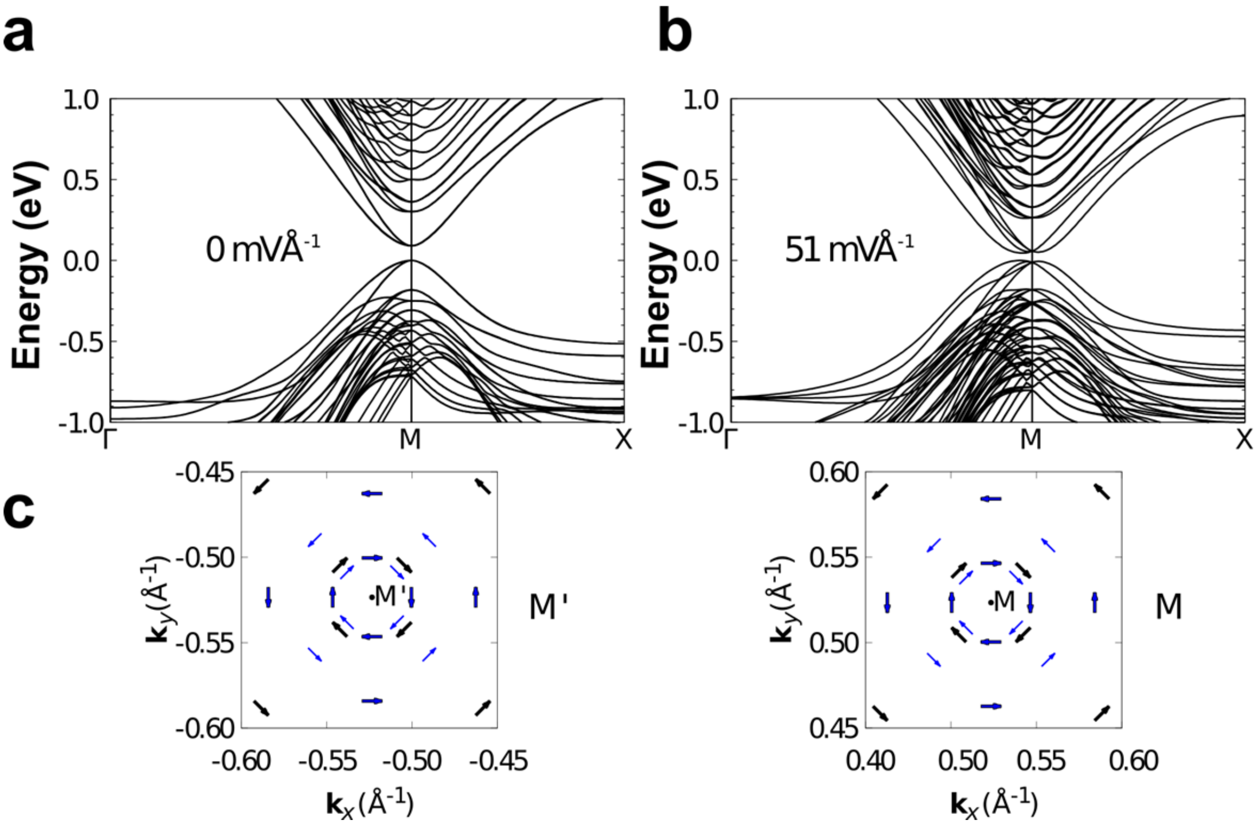

Supplementary Figure 5 | Band structure of lead sulfide nanosheets with Rashba splitting.

a, Without an external electric field, the band structure does not show Rashba-spin splitting along the Γ-M-X path. **b,** With an external electric field, (strength: 51 mV$Å^{-1}$) Rashba-spin splitting occurs at the M point in the valence band as well as in the conduction band. The valence-band maximum was set to 0 eV. **c,** Spin texture of the two highest valence bands, 0.044 eV below the valence-band maximum (black: highest filled band, blue: second highest filled band) at the M (right) and the M' point (left). A typical spin texture for Rashba-spin splitting can be observed[1]. The length of the arrows is normalized. Along the Γ-M path, the highest and second highest filled bands split in energy, while along the M-X path, they do not.

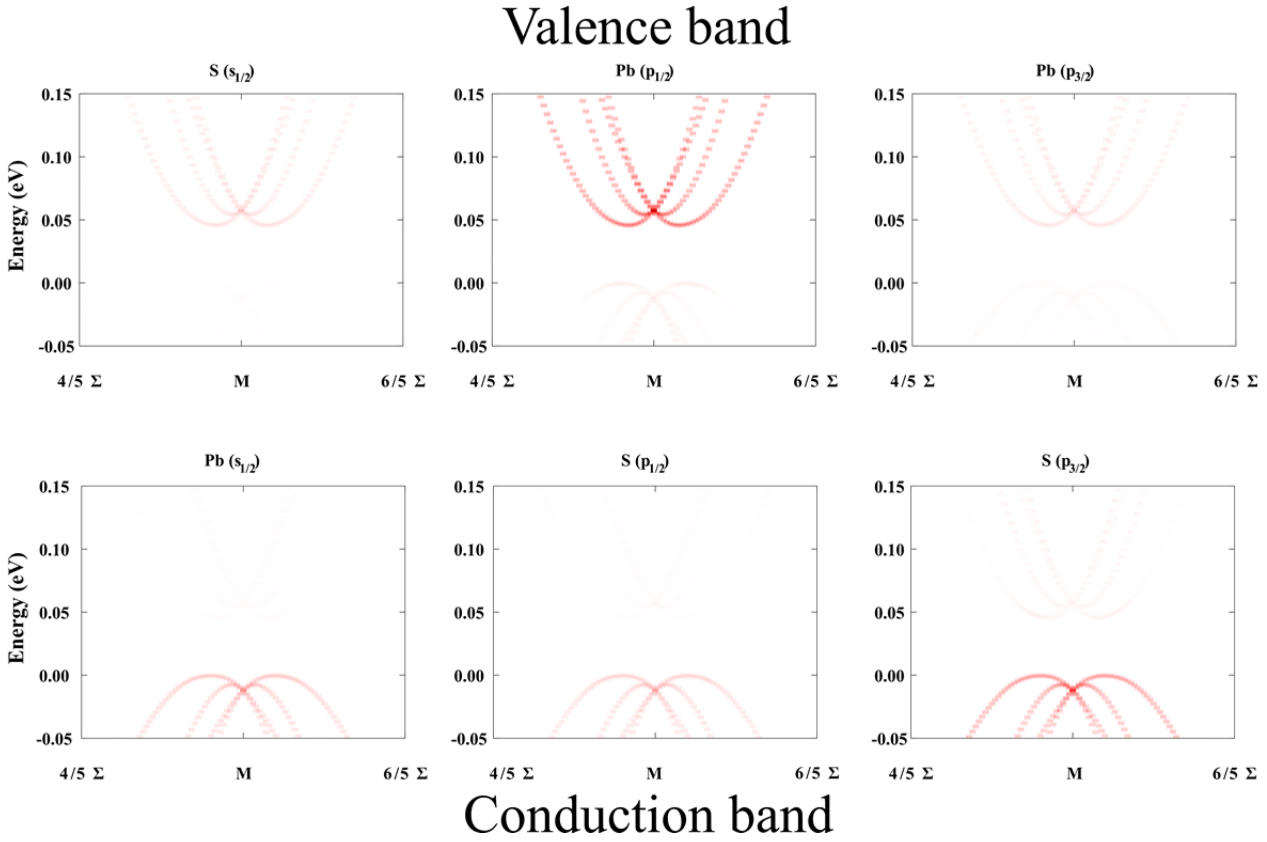

Supplementary Figure 6 | Projected density of states resolved on several k-points in the vicinity of the band gap.

The projected density of states was summed over all atoms for the corresponding orbital (E=51 mVÅ^{-1}). The valence band predominantly consists of the $p_{3/2}$ orbitals of sulphur (S), while the conduction band mainly consists of the $p_{1/2}$ orbitals of lead (Pb). The intensity of the colour implies the strength of the contribution of the corresponding orbital.

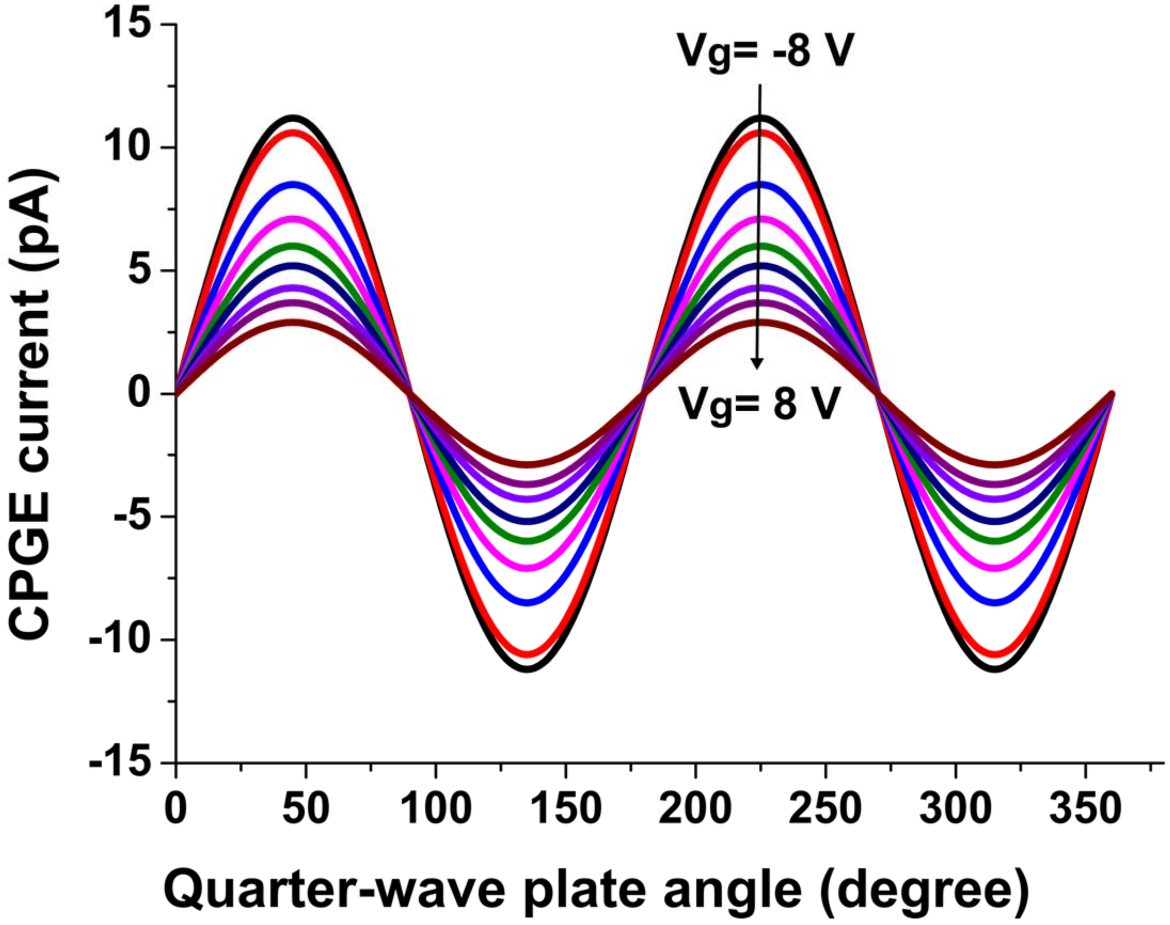

Supplementary Figure 7 | Extracted gate dependency for the magnitude of the circular photo-galvanic current.

By decreasing the back-gate voltage from 8 V to -8 V (in steps of 2 V), the absolute magnitude of the generated CPGE can be increased. Modification of the bands splitting as well as the recombination rate is the reason for such a tendency. By altering the angle of the quarter-wave plate, oscillations with period of 180° are detected.

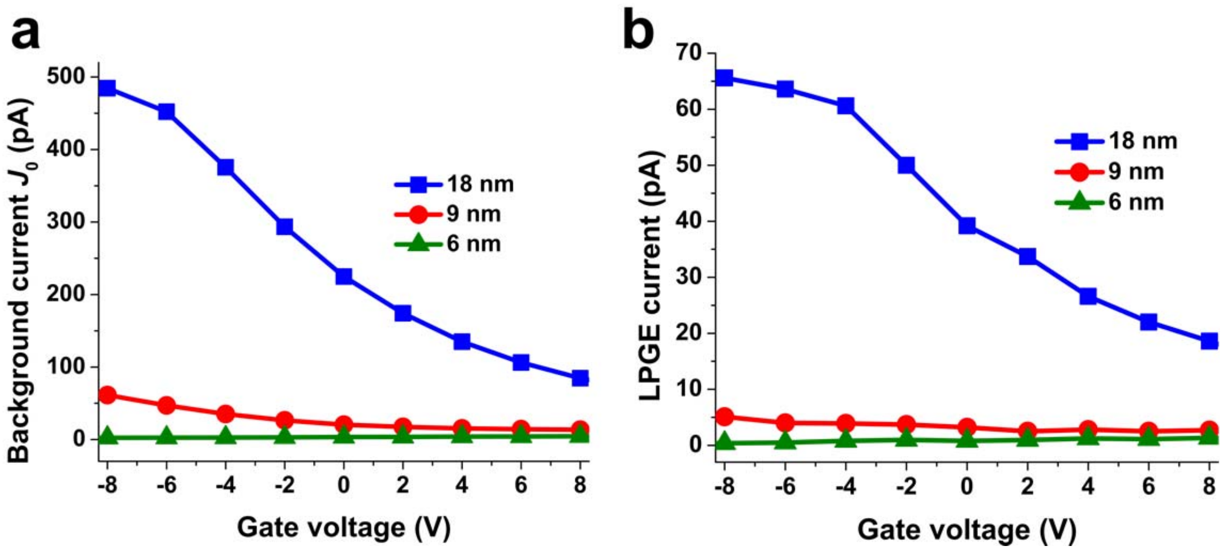

Supplementary Figure 8 | Spin-independent properties of the nanosheets with different thicknesses.

a, Background current J_0 for the investigated devices as a function of the back-gate voltage. Larger channel sizes as well as smaller band gaps for thicker sheets can lead to the generation of higher background currents. Modification of the band alignment by changing the gate voltage also tunes the background current for each thickness. **b,** Variation of the linear photo-galvanic (LPGE) current by changing the nanosheet thickness. By changing the circular polarization of the light, the spin-independent photocurrent can be modulated. Comparable with the background current, this component can be also tuned by the thickness or by the back-gate voltage.

Supplementary References

1. Manchon, A., Koo, H. C., Nitta, J., Frolov, S. M., Duine, R. A., New perspectives for Rashba spin–orbit coupling. *Nature Materials* ***14***, 871–882 (2015).
